# A nomogram model for predicting malnutrition among older hospitalized patients with type 2 diabetes: a cross—sectional study in China

**DOI:** 10.1186/s12877-023-04284-4

**Published:** 2023-09-15

**Authors:** Qian Ran, Xili Zhao, Jiao Tian, Siyuan Gong, Xia Zhang

**Affiliations:** 1 Department of Endocrinology, Jiangnan Campus, the Second Affiliated Hospital of Chongqing Medical University, Tianwen Street, Nanan District, Chongqing, 401336 People’s Republic of China; 2 Department of Neurology, the Second Affiliated Hospital of Chongqing Medical University, Chongqing, 401336 People’s Republic of China; 3 Department of Endocrinology, Chongqing City Hospital of Traditional Chinese Medicine, Chongqing, 400011 People’s Republic of China

**Keywords:** Nomogram, Malnutrition, Older, Type 2 diabetes, Risk factors, Prediction model

## Abstract

**Background:**

Malnutrition remains a pervasive issue among older adults, a prevalence that is markedly higher among those diagnosed with diabetes. The primary objective of this study was to develop and validate a risk prediction model that can accurately identify instances of malnutrition among elderly hospitalized patients with type 2 diabetes mellitus (T2DM) within a Chinese demographic.

**Methods:**

This cross-sectional study was conducted between August 2021 and August 2022, we enrolled T2DM patients aged 65 years and above from endocrinology wards. The creation of a nomogram for predicting malnutrition was based on risk factors identified through univariate and multivariate logistic regression analyses. The predictive accuracy of the model was evaluated by the receiver operating characteristic curve (ROC),the area under the ROC (AUC), the concordance index (C-index), and calibration curves.

**Results:**

The study included a total of 248 older T2DM patients, with a recorded malnutrition prevalence of 26.21%. The identified critical risk factors for malnutrition in this cohort were body mass index, albumin, impairment in activities of daily living, dietary habits, and glycosylated hemoglobin. The AUC of the nomogram model reached 0.914 (95% CI: 0.877—0.951), with an optimal cutoff value of 0.392. The model demonstrated a sensitivity of 80.0% and a specificity of 88.5%. Bootstrap-based internal verification results revealed a C-index of 0.891, while the calibration curves indicated a strong correlation between the actual and predicted malnutrition risks.

**Conclusions:**

This study underscores the critical need for early detection of malnutrition in older T2DM patients. The constructed nomogram represents a practical and reliable tool for the rapid identification of malnutrition among this vulnerable population.

## Background

Several populous nations, including China, Brazil, India, and Russia have been reported to have entered the demographic aging phase [1], although the rate of population aging varies, projections estimate that the ratio of adults over 65 years to the working population will nearly double in the next 40 years [2]. Coinciding with socioeconomic advancement, lifestyle changes, and an accelerated aging process, the global prevalence of diabetes is also increasing. Currently, around 463 million people worldwide are diagnosed with diabetes mellitus (DM), and this number is anticipated to reach 578.4 million by 2030 [3]. And China alone is home to a quarter of the world's older diabetic population, a trend that is predicted to rise [4], similar increases in the prevalence of older adults with diabetes are also expected in the United States and Japan in the forthcoming decades [5, 6]. These circumstances will undoubtedly impose substantial burdens on regulatory authorities, economies, and healthcare systems [7].

The diminished functionality and metabolic disturbances induced by insulin resistance in older diabetic individuals invariably escalate the risk of malnutrition [8, 9]. Prior studies have substantiated that the prevalence of malnutrition among older patients with type 2 diabetes mellitus (T2DM) ranges from 18.5% to 31.0% [10, 11], and a survey conducted among community-dwelling in China revealed a malnutrition rate of 38.2% among this population [12]. Additionally, Saintrain et al [13] have revealed that malnourished older persons with T2DM were hospitalized an average of 4 days longer than their well-nourished counterparts, and malnutrition is regarded as a strong predictor of adverse health outcomes like frailty, cognitive impairment and even mortality, all of which hinder these individuals' return to normal life and obviously diminish their quality of life [1, 14].

A risk prediction model employs mathematical equations to estimate the probability of an individual currently having a disease or experiencing a specific outcome in the future [15]. With the evolution of precision medicine paradigm, risk prediction models are gaining popularity in clinical practice, and nomograms are commonly utilized for model representation [16]. Previous researches have constructed nomogram models of malnutrition in fields such as oncology and organ transplantation [17, 18], however, these findings are not directly applicable to older T2DM patients. To our knowledge, Sanz et al [19] have demonstrated that gender, age, diabetic complications and albumin are the primary factors connected with malnutrition in older T2DM patients. Notwithstanding all this, the incidence and risk factors of malnutrition in Chinese older T2DM patients remain largely undefined and warrant further investigation.

Hence, this study aimed to incorporate the above potential contributors to malnutrition, investigate reliable indicators, and develop a risk prediction model for malnutrition in older hospitalized T2DM patients. Such a nomogram model can assist clinical practitioners in identifying individuals at high risk of malnutrition and formulating targeted preventive measures.

## Methods

### Setting and participants

**Fig. 1:**
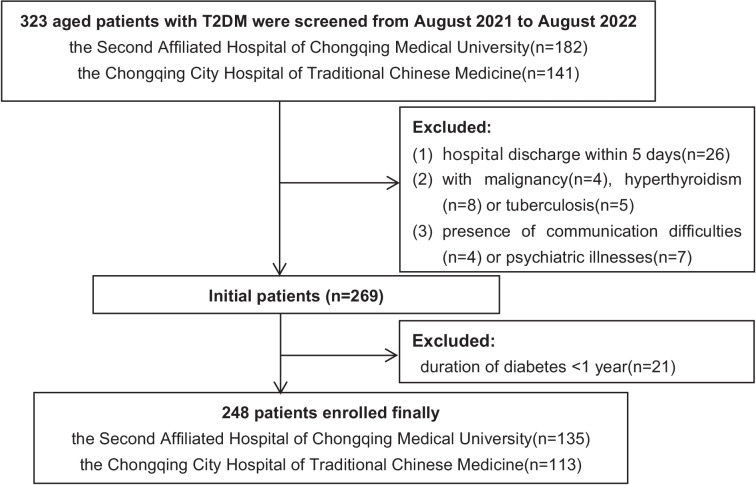
Flow diagram of the study population

This multicenter, cross-sectional study was conducted from August 2021 to August 2022. Patients were recruited from the endocrinology wards of the Second Affiliated Hospital of Chongqing Medical University and the Chongqing City Hospital of Traditional Chinese Medicine. Eligible participants met the following criteria: 1) diagnosed with T2DM for over 1 year [20], 2) aged 65 years or older, and 3) provided informed consent, either personally or through their caregivers. Exclusion criteria included: 1) hospital discharge within five days, 2) concurrent diagnoses of malignancy, hyperthyroidism, or tuberculosis, and 3) presence of communication difficulties or psychiatric illnesses. The recruitment process is illustrated in Fig. 1. This study received approval from the Ethics Committee of the Second Affiliated Hospital of Chongqing Medical University (2022–135).

### Data collection

#### Demographic data and Biochemical measurements

The collected demographic data comprised gender, age, history of smoking and drinking, marital status, comorbidities, diabetes course, educational level, personal monthly income, dietary habits, complications of diabetes, length of hospitalization, treatment and activities of daily living (ADL). The Barthel Index (BI) was utilized to evaluate ADL, with the scoring scale of 0 to 100, higher scores indicated lesser dependency [21].The scores were categorized into no dependency (100 points), mild dependency (61–99 points), moderate dependency(41–60 points), and severe dependency (≤ 40 points). All information was gathered by the diabetes specialist nurses upon patients' admission. Biochemical measurements, such as C-reactive protein (CRP), white blood cell (WBC), albumin (ALB), triglycerides (TG),high-density lipoprotein (HDL), glycosylated hemoglobin (HbA1c), hemoglobin (Hb), and glomerular filtration rate (GFR) were obtained from blood samples taken within 24 h’ admission. All tests were conducted in accordance with clinical guidelines by trained technicians at the hospital's central laboratory.

#### Anthropometric measurements

Anthropometric measurements were performed in the morning with patients wearing standard uniforms and without shoes. Calibrated scales and stadiometer were used to record weight and height. Body Mass Index (BMI) was calculated as weight (kg) divided by height (m) squared (kg/m^2^) and was categorized as underweight (< 18.5kg/m^2^), normal (18.5–23.9kg/m^2^) and overweight (≥ 24kg/m^2^) [22]. Patients with mobility issues were weighed in their wheelchairs, with their height provided by their caregivers. A single trained investigator performed all anthropometric measurements.

#### Nutritional Screening

Upon admission, all participants underwent Mini Nutritional Assessment Short Form (MNA-SF) [23], a validated screening tool designed specifically for the older population. This tool comprises six items with a scoring range of 0 to 14. MNA-SF scores are typically divided into three categories: normal nutritional status (12–14 points), risk of malnutrition (8–11 points), and malnourished (0–7 points). However, for the purposes of our study, we further simplified these into two categories: normal nutritional status (> 11 points) and malnourished (≤ 11 points) [24].

### Statistical analysis

We employed the Shapiro–Wilk test to examine the normality of continuous variables. Variables with a normal distribution were described by mean ± standard deviation, and those with a non-normal distribution were described by median and interquartile range. Categorical variables were reported as frequencies and percentages. The Student's t-test, Mann–Whitney U test, Chi-square test, or Fisher's exact test were adopted as appropriate to compare differences between groups. Subsequently, all variables with *P* < 0.05 in univariate analysis were incorporated into a multivariate stepwise logistic regression analysis using the Forward LR method, from which a nomogram was constructed. The predictive performance of this model was assessed via calibration curve and concordance index (C-index) [25]. Higher C-index values, which can range from 0 to 1, suggest better model discrimination. Internal validation of the model was performed using a Bootstrapping technique with 1000 repetitions. The receiver operating curve (ROC) was depicted, and the area under the ROC (AUC), which equals the C-index, was computed to evaluate the predictive accuracy of the nomogram. Statistical significance was set at a two-tailed *P* < 0.05. All analyses were performed using IBM SPSS 26.0 software (Armonk, NY, USA) and R 4.1.2 software (https://www.r-project.org/).

## Results

### Participants’ characteristics

A total of 248 older patients with T2DM, ranging from 65 to 93 years with a median age of 70 (interquartile range, 10) years were included in the study. Among these, 153(61.69%) were male and 95 (38.31%) were female. Notably, 65 patients (26.21%) were malnourished, while 183 (73.79%) were not. The results of the univariate analysis showed that age (*P* = 0.037), BMI (*P* < 0.001), dietary habits(*P* < *0.001*), ADL(*P* < *0.001*), WBC(*P* = 0.048), ALB(*P* < *0.001*), TG (*P* = 0.027), and HbA1c(*P* < *0.001*) were detected to be statistically related to the nutritional status of T2DM older patients. More characteristics for participants were presented in Table 1.

**Table 1:** Characteristics of T2DM older patients with or without malnutrition (*n* = 248)

Variables	Normal	Malnutrition	*P value*
	**(** ***n*** ** = 183)**	**(** ***n*** ** = 65)**	
Gender (n, %)			0.573
Male	111 (60.66)	42 (64.62)	
Female	72 (39.34)	23 (35.38)	
Age (n, %)			**0.037**
65–74 years	132 (72.13)	40 (61.54)	
75–84 years	48 (26.23)	20 (30.77)	
≥ 85 years	3 (1.64)	5 (7.69)	
BMI (n, %)			**< 0.001**
< 18.5 kg/m^2^	20 (10.93)	15 (23.08)	
18.5–23.9 kg/m^2^	51 (27.87)	38 (58.46)	
≥ 24 kg/m^2^	112 (61.20)	12 (18.46)	
Smoking (n, %)			0.941
Yes	100 (54.64)	48 (73.85)	
No	83 (45.36)	17 (26.15)	
Drinking (n, %)			0.860
Yes	95 (51.91)	40 (61.54)	
No	88 (48.09)	25 (38.46)	
Marital status (n, %)			0.506
Married	139 (75.96)	52 (80.00)	
Single/divorced	44 (24.04)	13 (20.00)	
Comorbidities (n, %)			0.533
≥ 3Types	115 (62.84)	38 (58.46)	
< 3Types	68 (37.16)	27 (41.54)	
Diabetes course,Years(median, IQR)	12 (6–20)	12 (6.5–19)	0.665
Educational level, (n, %)			0.616
Elementary school	46 (25.14)	19 (29.23)	
Middle school	63 (34.43)	23 (35.38)	
High school	42 (22.95)	10 (15.38)	
College and above	32 (17.49)	13 (20.00)	
Personal monthly income (n, %)			0.517
< 3000 CNY	75 (40.98)	30 (46.15)	
3000–5000 CNY	65 (35.52)	18 (27.69)	
> 5000 CNY	43 (23.50)	17 (26.15)	
Dietary habits (n, %)			**< 0.001**
Regular^a^	100 (54.64)	19 (29.23)	
Irregular	83 (45.36)	46 (70.77)	
Complications of diabetes(n, %)			0.780
≥ 3 Types	74 (40.44)	25 (38.46)	
< 3Types	109 (59.56)	40 (61.54)	
Length of hospitalization (n, %)			0.067
5–9 days	130 (71.04)	38 (58.46)	
10-14 days	47 (25.68)	21 (32.31)	
≥ 15 days	6 (3.28)	6 (9.23)	
Treatment (n, %)			0.182
Oral	50 (27.32)	15 (23.08)	
Subcutaneous injection	17 (9.29)	2 (3.08)	
Insulin pump	64 (34.97)	31 (47.69)	
Combined	52 (28.42)	17 (26.15)	
ADL (n, %)			**< 0.001**
no dependency	82 (44.81)	11 (16.92)	
mild dependency	75 (40.98)	13 (20.00)	
moderate dependency	15 (8.20)	19 (29.23)	
severe dependency	11 (6.01)	22 (33.85)	
CRP ≥ 10 mg/L (n, %)			0.235
Yes	139 (75.96)	54 (83.08)	
No	44 (24.04)	11 (16.92)	
WBC ≥ 10 × 10^9^/L (n, %)			**0.048**
Yes	30 (16.39)	18 (27.69)	
No	153 (83.61)	47 (72.31)	
ALB ≥ 35 g/L (n, %)			**< 0.001**
Yes	92 (50.27)	4 (6.15)	
No	91 (49.73)	61 (93.85)	
TG ≥ 1.7 mmol/L (n, %)			**0.027**
Yes	70 (38.25)	15 (23.08)	
No	113 (61.75)	50 (76.92)	
HDL < 1.29 mmol/L(n, %)			0.159
Yes	122 (66.67)	37 (56.92)	
No	61 (33.33)	28 (43.08)	
HbA1c ≥ 7% (n, %)			**< 0.001**
Yes	66 (36.07)	46 (70.77)	
No	117 (63.93)	19 (29.23)	
Hb,g/L ≥ 120(male)/110(female) (n, %)			0.155
Yes	155 (84.70)	50 (76.92)	
No	28 (15.30)	15 (23.08)	
GFR (n, %)			0.179
< 60 mL/min	40 (21.86)	21 (32.31)	
60-89 mL/min	91 (49.73)	25 (38.46)	
≥ 90 mL/min	52 (28.41)	19 (29.23)	

*Abbreviations*: *BMI* Body mass index, *IQR* interquartile range, *CNY* Chinese Yuan, *ADL* activities of daily living, *CRP* C-reactive protein, *WBC* white blood cell, *ALB* albumin, *TG* triglycerides, *HDL* high-density lipoprotein, *HbA1c* glycosylated hemoglobin, *Hb* hemoglobin, *GFR* glomerular filtration rate

^a^be on time for meals at least 5 days a week lasting for more than 3 months

### Risk factors for malnutrition in older patients with T2DM

The final multivariate logistic regression analysis demonstrated that the ALB (*P* < 0.001), HbA1c (*P* < 0.001), ADL (*P* < 0.05), BMI(*P* < 0.05)and regular dietary habits (*P* = 0.044) were independent risk factors for malnutrition in older T2DM patients (Table 2).

**Table 2:** Association between risk factors and malnutrition in multivariate logistic regression

Variables	***B***	***SE***	***P value***	***OR***	95%***CI***
ALB					
<35g/L	Reference				
≥35g/L	-2.945	0.628	<0.001	0.053	0.015-0.180
HbA1c					
<7%	Reference				
≥7%	1.570	0.434	<0.001	4.804	2.053-11.243
ADL					
no dependency	Reference				
moderate dependency	0.219	0.733	0.025	1.245	0.296-5.237
mild dependency	-0.721	0.670	0.013	0.486	0.131-1.807
severe dependency	-1.522	0.710	0.032	0.218	0.054-0.878
BMI					
<18.5kg/m^2^	Reference				
18.5-23.9kg/m^2^	-0.446	0.546	0.014	0.640	0.220-1.867
≥24 kg/m^2^	-2.844	0.624	<0.001	0.058	0.017-0.198
Dietary habits					
Irregular	Reference				
Regular^a^	-0.847	0.420	0.044	0.429	0.188-0.977

*Abbreviations*: *SE* Standard error, *OR* odds ratio, *CI* confidential interval, *ALB* albumin, *HbA1c* glycosylated hemoglobin, *ADL* activities of daily living, *BMI* Body mass index

^a^be on time for meals at least 5 days a week lasting for more than 3 months

### Development and validation of nomogram

**Fig. 2:**
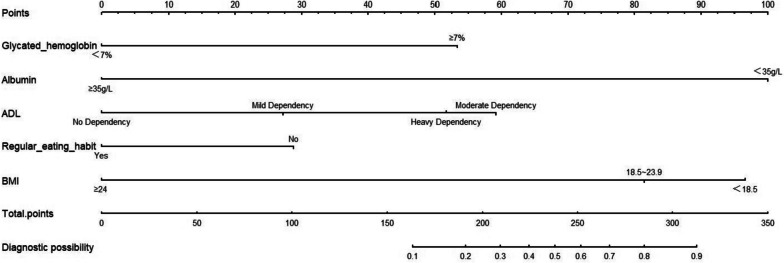
Nomogram of the risk prediction model for malnutrition in older T2DM patients

A nomogram model, constructed based on multivariate logistic regression analysis, was developed, which included five variables as shown in Fig. 2. Draw a line straight upward to the points axis to determine the score of each variable at each level, then calculated a total score of these predictors. Locate the final sum on the total points axis and draw a line straight down to indicate the malnutrition probability of patients. For example, a 68-year-old patient with T2DM, had an HbA1c of 6.5% (0 points), ALB of 34.2 g/L (100 points),ADL score of 80 (mild dependency, 27 points), regular eating diet (0 points), and BMI of 23.3 kg/m^2^ (81 points). The cumulative score for each predictor was 208, and the corresponding risk probability for malnutrition was approximately 29.7%.

**Fig. 3:**
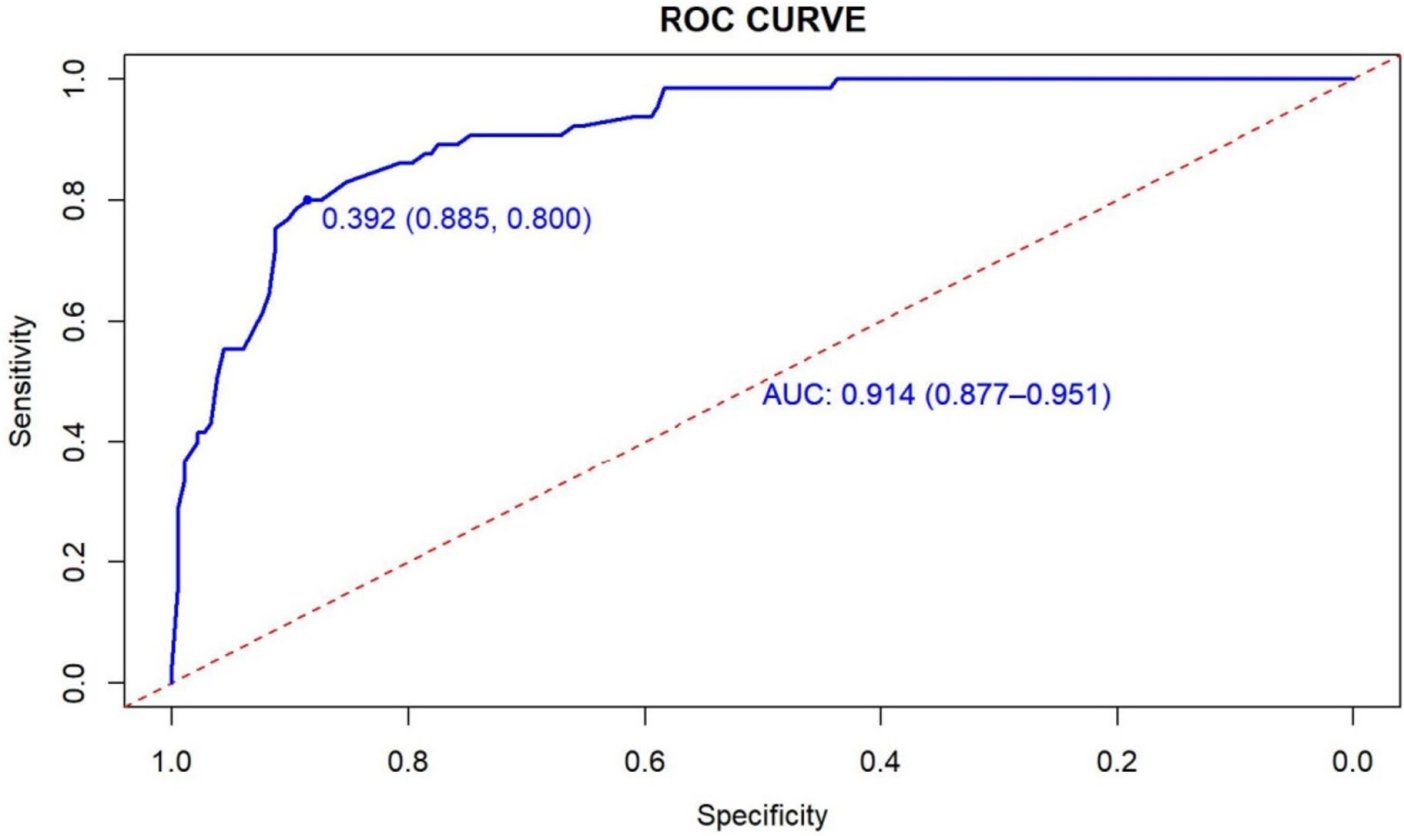
ROC curve of the nomogram

**Fig. 4:**
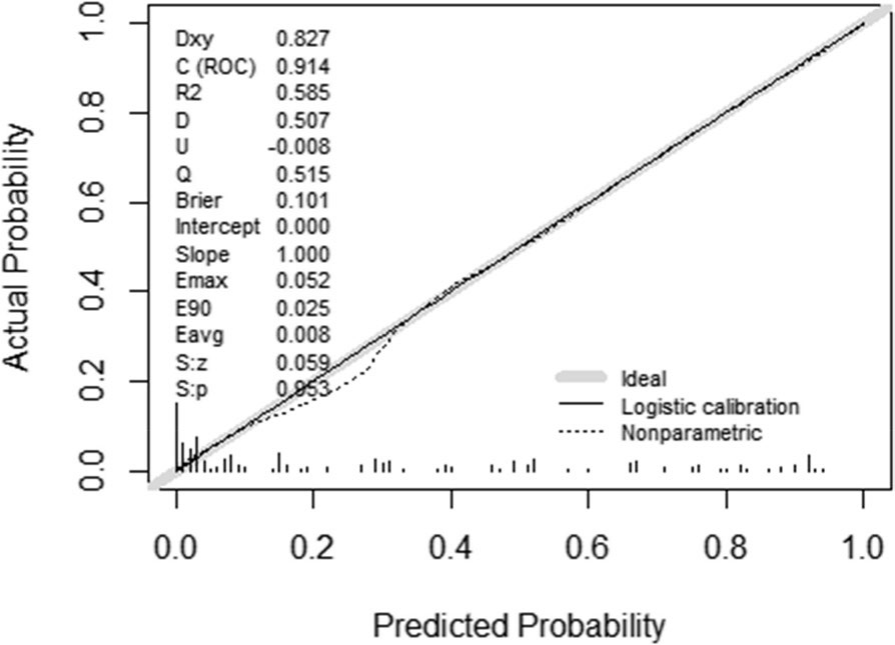
Calibration curve of the nomogram

The AUC of the nomogram was 0.914 (95%*CI*: 0.877–0.951), with a best cutoff value of 0.392. The sensitivity and specificity of the model were 80.0% and 88.5%, respectively (Fig. 3). Calibration plots (Fig. 4) demonstrated optimal agreement between the probability of malnutrition predicted by nomogram and the actual observed outcomes. Furthermore, the Bootstrap method was employed for validation, which yielded a C-index of 0.891, sensitivity of 90.0%, and specificity of 60.1%. In summary, the nomogram model demonstrated good predictive performance for malnutrition among older patients with T2DM.

## Discussion

Currently, research investigating malnutrition in China lags behind that in western countries, together with the fact that majority of scholars concentrate on the nutritional status of oncology and preoperative patients [26, 27], there is a dearth of studies that focus specifically on elderly patients with T2DM. In present study, we evaluated the nutritional status of older inpatients with T2DM by the MNA-SF and identified potential risk factors of malnutrition. Our results found that the occurrence of malnutrition in this patient group was 26.21%, which lies between 18.5% -38.2% reported in previous researches [10–12], reminding us the growing necessity of acknowledging and addressing malnutrition in this population. Besides, we developed a nomogram model which proved to be featured favorable efficiency in predicting malnutrition among older hospitalized patients with T2DM.

In our study, we took BMI < 18.5 kg/m^2^ as the reference group and found the risks of malnutrition in the BMI 18.5–23.9 kg/m^2^ and ≥ 24 kg/m^2^ groups were 0.640 and 0.058 times higher, respectively, than in the reference group. These results suggest that patients with higher BMI are less likely to develop malnutrition, which was unanimous with the conclusions drawn by Vural et al [28]. Studies showed patients with lower BMI exhibited more pronounced insulin resistance and have a poorer capacity for hepatic glycogen storage, while patients with higher BMI present more favorable metabolic profiles [29, 30]. However, it is worth noting that even overweight or obese older T2DM patients are vulnerable to malnutrition, mainly due to their lower basal metabolic rate, changes in body composition, and decreased appetite resulting from reduced physical activity and sensory function [13]. Furthermore, BMI-calculated as weight (kg) divided by height squared (m^2^), which might be overestimated in older subjects since the height in these individuals probably be shortened. Consequently, relying solely on BMI to assess nutritional status can be misleading.

Our results clearly highlight the risk of malnutrition in older T2DM patients with HbA1c > 7% was 4.804 times higher than those with HbA1c < 7%.In other words, HbA1c is negatively correlated with the patient's nutritional status, which means that good glycemic control may be realized at the expense of malnutrition. High HbA1c often means poor glycemic control, when patients are chronically hyperglycemic, the intensified metabolic disorders will result the synthesis of protein and other energy-supplying substances reduced significantly [31]. Moreover, chronic hyperglycemia can disrupt the balance of intestinal microflora, thereby impairing nutrient absorption from consumed food [32]. However, another published literature revealed that the HbA1c could be extended to 8.0% in malnourished patients [33]. In view of this, healthcare providers should set individualized HbA1c goals for patients and schedule regular reviews to adjust glycemic management plans timely.

In present study, we divided ADL into four levels and ultimately found the better the ADL, the lower incidence of malnutrition. A prospective, observational study carried out by Liu et al [10] in western China reported a similar finding, showing that dependence on ADL was associated with malnutrition (OR = 11.6, 95%CI: 5.10–26.5). It’s generally acknowledged that malnutrition and functional capacity are interrelated [34], this may be because individuals dependent on ADL require assistance to carry out daily activities, resulting in decreased physical activity and potential sedentariness, which, along with a lower basal metabolic rate, could lead to a loss of appetite. Besides, Naseer et al [35] supported that the survival rate of patients at risk of both malnutrition and ADL dependence (18.7%) was lower than the risk of malnutrition (37.8%) and ADL dependence (35.0%) alone. This suggests ADL dependence could exacerbate the consequences of malnutrition, thus highlighting the importance of delaying ADL dependence in older adults to improve their nutrition status and survival rates.

The findings also revealed a statistically significant correlation between ALB and malnutrition (*P* < 0.001), suggesting that patients with higher ALB presented lower probability of malnutrition. A retrospective study involving 439 T2DM adults showed variation in malnutrition degrees among patients with differing ALB levels [36]. More importantly, this study also confirmed that each unit increase in ALB is associated with a 63% reduction in mortality risk. Conventionally, an ALB concentration < 3.5 g/dL, which widely known as hypoalbuminemia, is regarded as a standard indicator of malnutrition. However, a meta-analysis indicted that this traditional definition may only identify the most severe risk of malnutrition rather than those at lower risk [37]. Accordingly, the relationship between ALB and malnutrition warrants further exploration.

Few studies have exploited the connection between dietary habits and malnutrition in older T2DM patients. In current study, we found that patients who maintain regular dietary habits are less likely to experience malnutrition. Regular dietary habits can replenish the energy and nutrients essential to support the body’s physiology and diseases consuming while avoiding additional burdens. Moreover, a recent systematic review has demonstrated that vegan and macrobiotic diets (a low-fat, high-fiber, high-complex carbohydrate, mainly vegetarian diet that was specifically designed for intensive treatment of T2DM patients [38]) were beneficial in glycemic control, improving insulin sensitivity and promoting weight loss [39]. Given the merits of a regulated diet, it’s imperative to implement multidisciplinary strategies to build a scientific nutritional management program. In conjunction with dietitians and rehabilitators, such a program could rectify poor dietary habits and optimize dietary structures.

This study is among the first to establish a prediction model for evaluating malnutrition risk in older T2DM patients. A nomogram was developed to visualize this model and facilitate the provision of risk probabilities to patients. In addition, we also analyzed factors such as biochemical indicators, lifestyle habits, and functional capacity. The final risk factors incorporated in the model are readily obtainable, cost-effective, and clinical applicable. However, this study still has some limitations. Firstly, this was a cross-sectional study and therefore cannot provide insights into causality, future research on this issue is needed to elucidate these relationships accurately. Secondly, as the study only included patients from two hospitals in China, the sample size and representativeness were insufficient, the applicability and generalizability of the model to broader populations need further validation. Finally, this study did not stratify the risk of malnutrition and therefore couldn’t establish graded intervention measures for patients at varying risk levels.

## Conclusions

In summary, our study identified that low BMI, low ALB, impaired ADL, irregular dietary habits, and high HbA1c are primary risk factors for malnutrition in elderly, hospitalized Chinese patients with T2DM. We also developed a predictive model for assessing the risk of malnutrition in this population. The results of the internal validation indicated that the constructed model exhibits commendable predictive performance. Our nomogram thus provides a convenient and reliable tool for predicting malnutrition in elderly patients with T2DM.

## Data Availability

The datasets used in this study are available from the corresponding author upon reasonable request.
